# Assessment of the benefits of seasonal influenza vaccination: Elements of a framework to interpret estimates of vaccine effectiveness and support robust decision‐making and communication

**DOI:** 10.1111/irv.12786

**Published:** 2020-09-03

**Authors:** Rosalind Hollingsworth, Clotilde El Guerche‐Séblain, Theodore Tsai, Yuri Vasiliev, Sam Lee, Helen Bright, Paula Barbosa

**Affiliations:** ^1^ Sanofi Pasteur Swiftwater PA USA; ^2^ Sanofi Pasteur Sanofi‐Aventis (Singapore) Pte. Ltd. Singapore Singapore; ^3^ Takeda Vaccines Cambridge MA USA; ^4^ St. Petersburg Research Institute of Vaccines and Sera Krasnoe Selo Russian Federation; ^5^ AstraZeneca Liverpool UK; ^6^ International Federation of Pharmaceutical Manufacturers and Associations Geneva Switzerland

**Keywords:** evaluative framework, influenza communications, Seasonal influenza, vaccination effectiveness, vaccination policy, vaccine recommendations

## Abstract

Systematic reviews and meta‐analyses confirm that influenza vaccination reduces the risk of influenza illness by between about 40% and 60% in seasons when circulating influenza stains are well matched to vaccine strains. Influenza vaccine effectiveness (IVE) estimates, however, are often discordant and a source of confusion for decision makers. IVE assessments are increasingly publicized and are often used by policy makers to make decisions about the value of seasonal influenza vaccination. But there is limited guidance on how IVE should be interpreted or used to inform policy. There are several limitations to the use of IVE for decision‐making: (a) IVE studies have methodological issues that often complicate the interpretation of their value; and (b) the full impact of vaccination will almost always be greater than the impact assessed by a point estimate of IVE in specific populations or settings. Understanding the strengths and weaknesses of study methodologies and the fundamental limitations of IVE estimates is important for the accuracy of interpretations and support of policy makers’ decisions. Here, we review a comprehensive set of issues that need to be considered when interpreting IVE and determining the full benefits of influenza vaccination. We propose that published IVE values should be assessed using an evaluative framework that includes influenza‐specific outcomes, types of VE study design, and confounders, among other factors. Better interpretation of IVE will improve the broader assessment of the value of influenza vaccination and ultimately optimize the public health benefits in seasonal influenza vaccination.

## 1 INTRODUCTION

Evidence of the impact of seasonal influenza vaccination programs is increasingly requested by governments or their Ministries of Health to rationalize their continued investment. In addition, in Europe, new European Medicines Agency (EMA) regulatory guidelines require that influenza vaccine Market Authorization Holders (MAHs) generate yearly, brand‐specific, influenza vaccine effectiveness (IVE) data.^1^ The Development of Robust and Innovative Vaccine Effectiveness project (DRIVE), which includes a number of influenza vaccine manufacturers, was designed to enhance the capacity for the estimation of IVE in Europe.^2^ A broader interest in the performance of influenza vaccines also demands a continuous investment from industry and academia into the assessment of IVE.

IVE estimates, however, are often discordant and a source of confusion, especially for those without extensive experience in vaccine trials design and statistics. Point estimates of seasonal IVE can vary greatly across seasons, populations, and health settings. Epidemiological factors account for some of these differences but often study design^3^ and the assessment of different outcomes make it difficult to compare studies within and across different seasons.^4^ The strengths and weaknesses of IVE study design have been extensively reviewed elsewhere.^5^, ^6^, ^7^, ^8^, ^9^, ^10^, ^11^ But methodological deficiencies in the evaluation of influenza vaccine effectiveness, and misinterpretation of the outcomes,^12^, ^13^ pose a serious challenge to the use of seasonal influenza vaccination as a public health tool.^14^ This paper reviews the critical considerations that should be made when interpreting IVE and proposes an evaluative framework to be used to interpret the outcomes of IVE studies as a basis for determining the full benefit of influenza vaccination.

### 1.1 Critical considerations for the review of IVE

Vaccine efficacy is estimated by experimental methods, in a well‐defined, controlled population, usually for the purpose of vaccine licensure and typically focuses on vaccine impact on laboratory‐confirmed influenza. Results of efficacy trials are specific to the conditions under study and therefore cannot be extrapolated to other situations, such as seasons or settings, where some of the associated conditions are likely to be different.

Vaccine effectiveness (VE) for influenza and other vaccines, on the other hand, is evaluated in real‐world settings and is typically estimated in observational studies, adjusting for potential confounding variables 5. Overall, recent effectiveness estimates of seasonal influenza vaccination against laboratory‐confirmed influenza (LCI) has ranged from about 40% to 60% when matched to circulating strains,^15^, ^16^, ^17^ and in individuals younger than 65 years of age typically ranges from 70% to 90%^18^ IVE is impacted by factors associated with the vaccine and by the risk of contracting influenza.^19^ Factors associated with the vaccine include the match between vaccine and circulating strains—lower IVE occurs when circulating strains of influenza virus drift from the strains included in the vaccine.^20^, ^21^, ^22^ IVE for seasonal influenza vaccination in any given year is highly contextual and variable by factors including include the match between vaccine and circulating strains (lower IVE occurs when circulating strains of influenza virus drift from the strains included in the vaccine^20^, ^21^, ^22^ ^),^ vaccine type, recipient age, setting (eg, inpatient/outpatient), and geography (see Table 1). Asymptomatic infections are most often not considered in IVE studies, in part because some study designs rely on symptoms for recruitment, even though asymptomatic infections may have an important role to play in transmission dynamics.^5^

**TABLE 1:** Recent systematic review and meta‐analyses estimates of influenza vaccine effectiveness

	Outcome	Group	Parameter of estimate	All types/ subtypes	H1N1pdm09	B	H3N2	H3N2 variant	Season/ Reference	Method
Outpatient setting	Laboratory‐confirmed influenza	All	VE		61%	54%	33%	23%	2004‐2015^16^	All observational
			95% CI		57, 65	46, 61	26, 39	2, 40		
			I^2^		0.0	61.3	44.4	55.6		
		>60 y	VE		62%	63%	24%			
			95% CI		36, 78	33, 79	‐6, 45			
			I^2^		0.0	0.0	17.6			
		≥65 y	VE	25%					2007‐2016^21^	Case‐control + cohort
			95% CI	6, 40						
			I^2^	0.0						
Hospital setting	Laboratory‐confirmed Influenza hospitalization	All	VE	41%	48%	38%	37%		2010‐11 to 2014‐15^22^	TN case‐control studies
			95% CI	34, 48	37, 59	23, 53	24, 50			
		18‐64 y	VE	51%						
			95% CI	44, 58						
		≥65 y	VE	37%			43%	14%		
			95% CI	30, 44			33, 53	‐3, 30		
			VE	44%					up to 2014^23^	
			95% CI	23, 60						
			VE	14%					2007‐2016^21^	Case‐control + cohort
			95% CI	7, 21						
			I^2^	19.2						

IVE for seasonal influenza vaccination in any given year is highly contextual and variable by type, age, setting (eg, inpatient/outpatient), and geography. Table 1 exemplifies how confusing IVE may be for policy makers when trying to ascribe a value to seasonal influenza vaccination, in the absence of proper context. It shows reported values of IVE between 14% and 63%, which policy makers might easily misinterpret in the absence of full context. Furthermore, even in relatively similar geographic settings estimates of IVE can be highly divergent. For instance, in the 2018‐19 season, overall interim IVE against H1N1pdm09 in the US was estimated at 47% (95% CI: 34 to 57), but at 72% in Canada (95% CI: 60 to 81).^24^

Similar discrepancies have been noted in European countries. For instance, in the 2015‐2016 season, data from vaccination among children in the I‐MOVE/I‐MOVE + multicenter case‐control study (11 continental European countries and Ireland) suggested there may not have been any protection against influenza B [−47.6% (95% CI: −124.9 to 3.1)], whereas in the same season in the UK IVE against influenza B in children was estimated at 56.3% (95% CI: −121.6 to 91.4).^25^

However, whatever the magnitude of the point estimate, the geography, season, setting, or age‐group evaluated, simply presenting the IVE estimate hides the considerable public health benefits of influenza vaccination programs. For example, in spite of a modest influenza vaccine effectiveness of 38%^26^ in the US 2017‐2018 season, seasonal influenza vaccination was estimated to have prevented 7.1 million illnesses, 3.7 million medical visits, 109,000 hospitalizations, and 8,000 deaths.^27^ In the 2020 season, the interim VE estimate in the United States indicates a 45% reduction in influenza illness associated with a medical visit which is sizeable given that during the previous decade, influenza caused an estimated 4.3‐21 million doctor visits and 140 000‐810 000 hospitalizations each year in the United States.^28^ Thus, even when VE is relatively low, the health and socio‐economic benefits of seasonal influenza vaccination can be appreciable.^5^, ^12^, ^29^

As such, it is important for policy makers to consider remember that IVE estimates alone are not a measure of the full benefits of seasonal influenza vaccination. Additional known heath impacts of seasonal influenza vaccination, such as reductions in exacerbations of specific underlying diseases such as chronic obstructive pulmonary disease (COPD), in asthmatic hospitalizations, in days of work/school lost, in nursing home epidemics, in the risk of complications such as myocardial infarction, stroke, and pneumonia, all contribute to the full public health impacts of vaccination.

In sum, the contextual variability of IVE calls for the use of a common evaluative framework to ensure the consistency of the assessment of IVE studies outcomes and to ensure that the strengths, weaknesses, and limitations of IVE data are fully appreciated by the reader.

### 1.2 Proposed elements of a framework for the assessment of seasonal influenza vaccine effectiveness

To account for differences in reported IVE estimates, WHO recommends that reporting of IVE studies include sufficient details on study participants, data collection, and analyses to enable readers to judge the validity of each study.^14^

With this in mind, and expanding on these ideas, we propose that an evaluative framework could be developed, which would include the following elements (as summarized in Table 2 and discussed below), to ensure the limitations of estimates of IVE, as an indicator of public health benefit, are fully appreciated and effectively communicated:

**TABLE 2:** Framework for the systematic assessment of IVE

	Elements of IVE studies to be systematically assessed	check list
1	Study outcomes (eg, laboratory confirmed influenza, influenza‐like illness) and setting (eg, outpatient, inpatient, medically attended)	Asymptomatic influenza
		Symptomatic influenza
		Medically attended influenza
		Hospitalization or severe illness from influenza
		Non‐communicable diseases (NCDs)
2	Study design	Experimental
		Observational
		Hybrid
3	Confounding factors	Viral/ epidemiological
		Sampling/ methodological

### 1.3 The outcomes measured

Several characteristics of the influenza virus, epidemiology, and vaccines create unique challenges for the evaluation of IVE and benefits of influenza vaccination programs. Most commonly, influenza vaccine efficacy and effectiveness are assessed against LCI. Ainslie et al^5^ report four primary outcomes of IVE studies, the last three being the most commonly assessed:

- asymptomatic influenza;
- symptomatic influenza;
- medically attended influenza; and,
- hospitalization or severe illness from influenza.

For each of these outcomes, IVE may differ. This is evidenced by the variability of IVEs against different endpoints, from different systematic reviews or meta‐analyses, as shown in Table 1.

IVE studies of asymptomatic influenza are important for assessing vaccination effectiveness against disease dynamics but are difficult and expensive to conduct. IVE studies of symptomatic influenza are important for assessing the impact of vaccination on the burden of disease, since even for those not seeking medical attention influenza is socially and economically burdensome. However, these studies are expensive and time‐consuming since they often require active surveillance and testing. Medically attended IVE studies are the most common because they can be the least logistically challenging, but they are prone to bias and often do not/cannot capture the effectiveness that influenza vaccine may have on other population outcomes. Likewise, IVE studies against hospitalizations and severe outcomes tend to show the highest IVE, but they are subject to selection bias because hospitalized individuals may not be representative of the entire population. It is important to note that studies usually focus on outcomes clearly related to influenza (influenza‐like illness, hospitalization for influenza/pneumonia), but ignore the broader impact of influenza and vaccines on outcomes where the role of influenza may be less apparent, such as exacerbations/destabilization of non‐communicable diseases (NCDs) (eg, COPD, diabetes) or as a trigger of serious events, such as myocardial infarction or stroke.

To recap, IVE is specific to an outcome, and each outcome assesses a different impact of vaccination. When evaluating IVE, it is important to be mindful of the outcomes reviewed and thus the relevance of the outcome to the population or setting when subsequently communicated.

## 2 STUDY DESIGN AND METHODOLOGY

Several types of study design are used to assess IVE. Within a same study type, definitions, methods, statistical analyses, and outcomes may vary. For this reason, a critical understanding of study design is a prerequisite for interpreting IVE, as each of these factors may impact IVE estimates.

### 2.1 Experimental, observational, and hybrid methods

Experimental methods, used for efficacy studies, in clinical trials, under ideal conditions, generally focus on the direct effect of the vaccine, and like for observational studies, results are season‐specific and not predictive of efficacy in subsequent seasons. The precisely defined inclusion criteria in randomized controlled trials (RCTs) limit the representativity of the outcomes. Under real‐world conditions, use of vaccines in a public health system, with high vaccination coverage, may have a much larger population impact than estimated in an RCT.

Observational methods assess effectiveness under real‐world conditions and across populations, and so effectiveness for a same vaccine may differ from efficacy. Observational studies are commonly used for post‐licensure surveillance. They may be used to evaluate several different impacts: the indirect effects of the vaccine from herd immunity; the overall public health value of the vaccination program; the duration of protection from vaccination; and the impact of vaccination on disease ecology, such as the impact on influenza strain circulation. They are less precise (wider confidence intervals) than experimental studies, but more practical (smaller samples size, shorter duration).

In observational studies, VE is estimated by rate ratios or hazard ratios of events occurring in vaccinated versus unvaccinated persons over time5. But these methods cannot control for bias to the same degree as prospective randomized clinical trials.^30^ However, observational studies can estimate the impact of vaccination programs on the entire exposed population.

In other words, experimental studies answer the binary question ‘does the vaccine work?’ (yes or no), whereas observational studies address ‘how well a vaccine works’.

A hybrid experimental‐observational method, referred to as “pragmatic clinical trial” (PCT)^31^ has also recently been used to estimate vaccine effectiveness. This study design investigates randomized groups prospectively, but measures endpoints from routinely collected data or vital statistics. The primary advantage of pragmatic/hybrid clinical trials is that they can be designed to be more reflective of real‐world vaccine experience, with research questions (such as outcomes, patients populations, and so on), that are more relevant to policy makers, clinical decision makers, and others as they seek to optimize immunization programs. This design is currently being used to compare the effectiveness against LCI of licensed egg‐based inactivated influenza vaccines against two other types of licensed vaccines (cell‐culture inactivated and recombinant).^32^

### 2.2 Strengths and weaknesses of study designs

The large number of study designs used to assess IVE underscores the imperfect nature of each.

#### 2.2.1 The randomized controlled trial (RCT) or Group Randomized Trial (GRT)

The RCT is the only design that controls for selection bias and confounding in the determination of causal association. A properly designed RCT controls for exposure and measures its effect with strong internal validity. The main weaknesses are (a) the limited transposability of the results to the broader community (external validity), given that participants may not be representative of the population, and (b) the size and associated costs of studies. Group or cluster randomization trials (GRT) can substitute for individual randomization, to reduce sample size and costs and avoid within group dependencies, such as from “herd immunity,” or to estimate an indirect effect of a vaccine.

#### 2.2.2 Cohort studies

Cohort studies, the gold standard design for estimating incidence rates, relative risks, and attributable risks, proceed in a logical sequence from exposure to outcome. They allow for hypotheses about causality of the exposure on the outcome and can provide an estimate of the VE. However, for influenza disease, at an attack rate of 5 to 10%, the duration and cost of studies can be prohibitive.

#### 2.2.3 Case‐control studies

Case‐control studies identify cases (outcome) and then ascertain exposure status. This design is the most appropriate for diseases with low incidence rates or with long duration between exposure and outcome incidence. The main advantages of case‐control studies are that they require a smaller sample size than cohort studies, they can be relatively inexpensive, and they can be relatively short in duration. They can also be used to assess VE in real time, from sentinel screening and surveillance. But since case‐control studies do not measure incidence rates, VE is estimated from the odds ratio. When the relative risk of exposure is small (<5%), the odds ratio can approximate the relative risk.

The main weaknesses of case‐control studies are that the exposure status is subject to recall bias, and the strength of causal association (odds ratio) is not as not as deterministic as the relative risk. Furthermore, accounting for confounding factors to match controls, requires knowledge of risk factors.

Two variants of case‐control studies, screening and case‐cohort studies, offer the advantage of selecting population‐representative controls by enrolling cases and non‐cases from a same cohort.

Over the past decade, the test‐negative design (TND) has emerged as the most popular and recommended method for estimation annual IVE5. In this design, VE is calculated as 100% × (1 ‐ odds ratio) for vaccination in influenza cases compared to vaccination in test‐negative controls. In this design, the study group is made up of patients seeking medical care for an acute respiratory illness and cases are defined as cases who test positive for influenza by reverse transcription polymerase chain reaction (RT‐PCR). This design has been found to yield valid estimates of VE in the source population under most scenarios.^17^

#### 2.2.4 Other designs

With large databases of patient registries, electronic health records, insurance data, web/ social media, or records of pharmaceutical products sales, observational data not collected under experimental conditions can be used to estimate “real‐world” effectiveness. Advantages and weaknesses of these estimates are mainly linked to the quality and completeness of the data collected.

A summary of the advantages and disadvantages of main study designs are summarized in Table 3.

**TABLE 3:** Summary of the advantages and disadvantages of the different study design for estimating influenza VE. For all the designs, study duration is for at least 1 influenza season

	Strengths	Weaknesses	Application to IVE studies
RCT	Strong internal validity; Avoids confounding bias; Demonstrates causal association.	Limited external validity; Patients are not representative of routine practice; Costly.	Efficacy, phase III or IV
GRT (or Cluster RT)	Simulate public health practice; Estimates direct and indirect benefit from influenza vaccination.	Implementation and identification of sites/groups can be long; Costly.	Efficacy or effectiveness, phase III or IV
Cohort	Temporal relationship exposure‐disease is clear; May feed several research projects.	Long duration from exposure to the outcome.	Effectiveness, phase IV
Case‐control (CC)	Quick to implement.	Temporal relationship exposure‐disease difficult to establish; Several potential biases: control selection, recall biases when collecting data; Cannot be used for new product.	Effectiveness, phase IV
Nested CC	Prospective study; No memory bias; Better control of biases than CC		Effectiveness, phase IV
CC – Sentinel Network	Real‐time VE estimation.	Heterogeneity in case definition and data between sites/countries; No distinction between vaccine type and brand.	Effectiveness, phase IV
Screening	Real‐time VE estimation.	Heterogeneity in case definition and data between sites/countries; Controls may not be representative of the population; No distinction between vaccine type and brand.	
Meta‐analysis	High level evidence.	Heterogeneity between studies; publication bias; Review limited by language.	Efficacy, effectiveness, phase IV

### 2.3 Confounders and other factors that influence IVE

Identifying factors that are strongly associated with vaccination and with disease risk is key to interpreting IVE estimates. At least two sets of factors play an important role in determining the likelihood of IVE against illness: 1) host characteristics (such as age, underlying health conditions, and level of pre‐existing immunity to circulating strains of influenza), and 2) vaccine characteristics, including the match between circulating stains of influenza virus and influenza vaccine strains.^33^ In years when vaccine strains are mismatched with circulating strains, IVE will be lower. These factors alone can explain most of the observed inter‐season variability in benefit.^21^

Several factors that confound comparisons between studies must be considered. The main factors that can confound IVE are identified in Table 4. Some of these are discussed below.

**TABLE 4:** Potential factors that can confound IVE

Viral/ epidemiological	Strain match—circulating strains can be genetically diverse, differing between seasons, geographic regions, and countries. Antigenic drift can occur after vaccine strain selection.
	Potential for adaptive mutations in HA during virus growth and passage in eggs or cell culture.
	Type/subtype‐specific effectiveness—predominant circulation of a subtype as effectiveness is highest for H1N1 and lowest for H3N2. Transmissibility and virulence of viruses can vary (from mild to severe disease).
	Vaccine‐type effectiveness—VE is brand‐specific as production methods (split virus, sub‐unit, etc) and processes (purification, inactivation, etc) differ between manufacturers.
Sampling/ methodological	Healthy user effect.
	Timing of enrollment into study during influenza season in relationship to vaccine uptake and incidence of influenza.
	Waning seroprotection
	Herd effects.
	Repeated vaccination.
	Vaccination history.
	Accuracy of link between record of immunization in databases and cases.

### 2.4 Intrinsic characteristics of the virus and its evolutions

#### 2.4.1 Strain match

IVE is dependent on the match between vaccine strains and circulating strains. IVE will be lower in years when there is a mismatch. Mismatches may occur in any season, in different geographic areas, when the predominance of a circulating strain changes, or if antigenic drift occurs after strain selection. The impact of strain mismatch is variable. For example, in adults vaccinated with trivalent inactivated vaccine, vaccination with a mismatched strain resulted in up to 13% decrease in VE when compared with vaccination with matched strains.^34^ In a hospital setting, the elderly may be particularly susceptible to a decrease in VE from strain mismatch.^35^ In a meta‐analysis, VE against H3N2 in hospitalized persons 65 years or older was 29% lower when strains were mismatched compared to matched strains.^36^ However, because of cross‐protection, vaccines containing a potentially mismatched strain may still have significant effectiveness.^34^, ^37^

#### 2.4.2 Virus growth in eggs and cell culture—potential for mutation

Generally, reassortant viruses must be adapted in eggs in order to produce high‐yield candidate vaccine viruses.^38^ During this process, mutations of influenza viruses may result in altered antigenicity. While antigenicity is continually tested, it can be difficult to accurately predict and these mutations may affect vaccine match with circulating strains. Vaccines are now available that are not manufactured using eggs. A licensed cell‐based manufacturing process uses Madin‐Darby Canine Kidney cells as the vehicle for replicating influenza virus. By avoiding the need for viral adaptation for growth in eggs, these vaccines may offer better protection than traditional, egg‐based influenza vaccines. In contrast to both egg‐ and cell‐manufactured vaccines, a licensed recombinant influenza vaccine manufacturing process does not involve viral growth in any medium, neither egg nor cell. Instead, recombinant vaccines are created synthetically. By avoiding the need for viral adaptation for growth in eggs, vaccines manufactured using alternative technology may offer better protection than traditional, egg‐based influenza vaccines. In the 2017‐2018 influenza season, a single amino acid mutation in egg‐adaptation was likely responsible for the estimated 25% vaccine effectiveness against the circulating H3N2 virus in adults in the United States. A study of over 1 million primary medical care encounters during the 2017/2018 season demonstrated, after allowing for many of confounding the factors noted above, an improved relative effectiveness of the cell‐manufactured vaccine of 36.2% overall.^39^ Age‐related differences were noted in this study. Other studies, however, suggested more modest improvements of around 10% (95% confidence interval [CI], 7%–13%) more effective.^40^

Additional data are needed to more clearly understand differences in performance of cell culture‐, recombinant‐, and egg‐manufactured vaccines, over multiple seasons, in different populations, and whether there may only be an advantage in specific epidemiological situations, such as seasons when A/H3N2 predominates.

#### 2.4.3 Virus type/subtype

Vaccine effectiveness is viral type/subtype specific. VE is typically highest for H1N1 and lowest for H3N2 (Table 1). Inactivated influenza vaccine is occasionally less effective against influenza A(H3N2) viruses (because of more frequent genetic and egg‐adapted changes leading to antigenic changes), and in elderly populations. Thus, in seasons where A(H3N2) is dominant there may be increased influenza morbidity and mortality, especially if antigenic drift contributes to reduced VE.^41^, ^42^, ^43^

VE for B types may be similar to H1N1 or slightly lower (see Table 1).

#### 2.4.4 Vaccine type/origin

Vaccines are produced by several manufacturers, and none are identical. Differences in production methods (split virus, sub‐unit, etc) and processes (purification, inactivation, etc) may mean that the nature of the vaccine itself may impact VE, making VE brand‐specific. The use of quadrivalent vaccines versus trivalent vaccine may also affect the IVE estimates since more strains (2 lineages of B) are covered by quadrivalent vaccines. The DRIVE project1 seeks to achieve high quality, brand‐specific effectiveness estimates for all influenza vaccines used in the EU each season. However, with frequent use of a number of different influenza vaccines in any given population, few vaccine registries and reliance on patient recall, developing valid brand‐specific IVE estimates will be challenging.

### 2.5 Sampling/ methodological confounding factors

#### 2.5.1 Healthy user effect

One of the main confounders in observational studies is the healthy user effect. This refers to a situation where patients in better health conditions are more likely to adhere to the annual recommendation for influenza vaccination. If not adjusted for comorbidities or health seeking behavior, the healthy user bias can overestimate IVE. Several approaches to control for this effect, such as by using propensity scores and other means, exist in the literature.

In a recent review of observational studies, Remschmidt et al^44^ found that 19 of 23 studies (83%) showed high risk of bias: 61% for confounding by indication, 9% for healthy user bias, and 13% for both forms of confounding/bias. Even after adjusting, residual confounding by healthy user effects was still present in the adjusted data. The authors concluded that the resulting estimates were still prone to healthy user bias for unspecific outcomes like all‐cause mortality.

#### 2.5.2 Timing of enrollment

The seasonality of influenza varies considerably around the world, ranging from well‐defined seasonal epidemics to year‐round circulation.^45^ For IVE studies, enrollment of study subjects should only be done during periods of influenza virus circulation in the study population, otherwise subjects will invariably test negative for influenza and be treated as non‐cases. This can lead to biased VE estimates.^14^, ^46^

Related to this is the time at which the estimate of IVE is made; mid‐season or end of season. Both low and high mid‐season IVE estimates may deter individuals from seeking vaccination when in both cases considerable benefits could still be obtained from vaccination. Furthermore, divergent results have been reported mid‐season vs. at the end of a season; for example, the interim IVE reported for the United States for the 2018‐2019 season was ultimately discordant with the end of season estimate of 29%.^47^ This may be linked to several factors including antigenic drift, changes in the dominance of viral type/subtypes, or lower depletion of susceptible. Until each of these contributing factors has been elucidated, the correct interpretation of interim IVE would be difficult to arrive at. Moreover, from a programmatic view point, there is no particular advantage of an interim IVE over a seasonal IVE.

#### 2.5.3 Waning seroprotection

Post‐vaccination antibody titers decline over time, and although titers usually remain at levels above those associated with protection for the duration of an influenza season,^48^, ^49^, ^50^ some degree of decline in VE may result from waning immunity. Several studies have reported that seasonal influenza vaccine protection becomes suboptimal beyond 4 months.^51^, ^52^, ^53^

A meta‐analysis of 14 studies found a significant decline in IVE for influenza virus subtype A/H3N2 and type B 91‐180 days after vaccination compared to 15‐90 days post‐vaccination, but the decline for H1N1 was not statistically significant.^54^ The analysis found that waning immunity, strain changes during the season, and herd immunity among controls could all bias estimates of IVE.

The above data are not sufficient to draw firm conclusions about the persistence of seroprotection over a defined period. Also, the evolution of influenza virus strains within the same season makes it difficult to distinguish waning vaccine‐induced immunity from decreasing match between the vaccine and circulating strains. A further confounder is that vaccine protection from influenza is not binary (all or nothing) but an odds ratio will not account for an incomplete protective effect of vaccination.^55^

#### 2.5.4 Herd effects

VE will vary with the vaccination coverage rate. This is because at higher levels of vaccination coverage, virus transmission will be interrupted and herd effects need to be considered, whereby the unvaccinated are protected by the reduction of disease transmission resulting from vaccination. With highly transmissible diseases, like measles (attack rate of approximately 90% in susceptibles^56^), herd effects can be very difficult to achieve. However, for influenza (attack rate of about 5% to 10% in adults, and 20% to 30% in children^57^), herd effects can be much easier to achieve and may be a factor in the estimation of VE where high vaccination coverage is achieved. In one study from the United States, LAIV vaccination of <20% of children reduced medically attended acute respiratory illness in adults by 8‐18%—a herd effect.^58^

### 2.6 Repeated vaccination

The concept of original antigenic sin has been demonstrated to occur in human influenza infection.^59^ This refers to a situation where a viral epitope varies slightly from an earlier infection, inducing an immune response to the epitope from the earlier infection rather than to the new epitope. This may result in an inadequate immune response to a new influenza strain. The same principle applies to vaccine epitopes, which may result in reduced vaccine effectiveness when new virus epitope circulate.

Studies of repeated vaccination across multiple seasons have suggested that VE may be influenced by more than one prior season. In the US, McLean et al^60^ found significantly higher VE for A/H3N2 and influenza B when there was no prior history of vaccination compared to persons with repeated vaccinations. In Europe, Rondy et al^61^ found that repeated vaccination reduced hospitalization VE against A/H1N1 but not against A/H3N2 or B. In immunogenicity studies, repeated vaccination has been found to blunt the hemagglutinin antibody response, particularly for H3N2.^62^, ^63^

On the other hand, a recent study from Canada investigating the associations between prior influenza vaccines and subsequent IVE during four consecutive seasons concluded that even when a negative impact on VE was observed, repeated vaccination was still more effective than not receiving seasonal vaccination.^64^ Therefore, repeated vaccination remains a subject of much discussion, and additional multi‐season clinical studies are needed to better understand whether repeat vaccination negatively impacts VE.

#### 2.6.1 Vaccination history

Vaccination history has become one of the latest confounders to come under scrutiny in test‐negative design studies. Vaccine protection from influenza is not binary (all or nothing) but an odds ratio will not account for an incomplete protective effect of vaccination.^55^ There are also questions about whether differences in estimates of IVE can be explained by factors like immune or vaccine history. For example, an unvaccinated individual may be afforded some protection from influenza due to prior exposure to influenza or previous vaccination history; thus, estimates of IVE are relative and not absolute measures of effectiveness.

#### 2.6.2 Accuracy of vaccination history

The accuracy of immunization records from clinical or administrative databases that are associated with cases and comparison groups is critical. Invalid immunization history may compromise the validity of a study, and reliance on patient recall may also confound estimates.

## 3 CONCLUSIONS

While the need to monitor vaccine performance by estimating IVE is recognized, no single methodology is perfect, and the headline‐grabbing point estimates communicated during and at the end of each season hide the considerable public health benefits of vaccination, even in years with modest vaccine effectiveness. Annual studies of IVE have limitations, are difficult to generalize, and, if considered in isolation, will not provide a comprehensive picture of the public health impact of influenza vaccines. However, development and implementation of a common evaluative framework may offer a consistent and objective approach to the assessment of IVE estimates, and help to ensure that the strengths and weaknesses of the data can be appreciated. We recommend that before VE estimates are used to support policy, they should be evaluated against the criteria outlined Table 2. Using a framework to support the development of vaccine policy will improve understanding of the impact of seasonal vaccination programs and thus may drive much‐needed urgency in influenza prevention efforts. Similar frameworks and approaches could also be applied to assess VE for other vaccines (eg, SARS‐CoV‐2).

## AUTHOR CONTRIBUTION

**Rosalind Hollingsworth:** Conceptualization (lead); Methodology (lead); Project administration (lead); Writing‐original draft (lead); Writing‐review & editing (equal). **Clotilde El Guerche Seblain:** Conceptualization (lead); Methodology (lead); Project administration (lead); Writing‐original draft (lead); Writing‐review & editing (equal). **Theodore Tsai:** Conceptualization (equal); Methodology (equal); Writing‐original draft (equal); Writing‐review & editing (equal). **Yuri Vasiliev:** Conceptualization (equal); Methodology (equal); Writing‐original draft (equal); Writing‐review & editing (equal). **Sam Lee:** Conceptualization (equal); Methodology (equal); Writing‐original draft (equal); Writing‐review & editing (equal). **Helen Bright:** Conceptualization (equal); Methodology (equal); Writing‐original draft (equal); Writing‐review & editing (equal). **Paula Barbosa:** Conceptualization (supporting); Methodology (supporting); Project administration (lead); Supervision (lead).

### Peer Review

The peer review history for this article is available at https://publons.com/publon/10.1111/irv.12786.
